# Moving Towards Acceptance and Values: A Qualitative Study of ACTforIBD Compared to IBD Psychoeducation

**DOI:** 10.1007/s10880-023-09999-5

**Published:** 2024-02-12

**Authors:** Kathryn Wilkin, Subhadra Evans, Leesa Van Niekerk, Daniel Romano, Matthew Fuller-Tyszkiewicz, Simon Knowles, Susan Chesterman, Leanne Raven, Antonina Mikocka-Walus

**Affiliations:** 1 School of Psychology, Deakin University, Geelong, Melbourne, VIC Australia; 2 School of Psychological Sciences, University of Tasmania, Churchill Avenue, Sandy Bay, Hobart, TAS Australia; 3 Department of Psychological Sciences, Swinburne University of Technology, Melbourne, VIC Australia; 4 Crohn’s & Colitis Australia, Melbourne, VIC Australia

**Keywords:** Acceptance and commitment therapy, Adult gastroenterology, Inflammatory bowel disease, Psychological distress, Qualitative

## Abstract

The current study explored perspectives of those with inflammatory bowel disease (IBD) and comorbid anxiety and/or depression on a hybrid acceptance and committment therapy (ACT) intervention, compared to an active control. This qualitative study was nested within a randomized controlled trial (RCT) where an experimental group received an 8-week blended delivery ACTforIBD intervention (four sessions telehealth, four sessions pre-recorded self-directed), while an active control group received a psychoeducation program of similar intensity. Semi-structured interviews were conducted post-intervention and at a 3-month follow-up. Themes were interpreted using reflexive thematic analysis. Twenty individuals participated; ten in each condition. Seven themes were constructed, including three shared themes between groups: *I Am Worth Advocating For, Present Moment Is My Biggest Ally,* and *Ambivalence About Self-Directed Modules*. Two themes were identified for the ACTforIBD group: *Symptoms Are Going to Happen* and *Moving Toward Values* while two themes identified from the ActiveControl group were: *Reset and Refresh* and *It’s Ok to Say No*. Acceptance and values modules from ACTforIBD were perceived as useful in reducing psychological distress for those with IBD, while the ActiveControl group felt their program affirmed existing effective coping strategies. Access to external resources for self-directed modules and networking may increase engagement with content long term.

## Clinical or Methodological Significance

This study explored perspectives of people living with IBD and comorbid anxiety and/or depression who took part in either an ACT intervention or an active control. Acceptance and values modules from the ACTforIBD intervention were seen as useful by participants in reducing psychological distress for those with IBD. Moreover, the ActiveControl group felt their program affirmed their existing effective coping strategies.

## Introduction

Inflammatory bowel disease (IBD) is a relapsing remitting condition, characterized by chronic inflammation of the gastrointestinal tract and includes Crohn’s disease and ulcerative colitis (Chang, 2020). IBD is thought to be a result of a dysregulated immune response to healthy intestinal bacteria and is influenced by both environmental and genetic factors (Malmborg & Hildebrand, 2016). Common physical symptoms of IBD include chronic pain or cramps, fatigue, recurring diarrhea or melaena, lethargy, and weight loss. Those affected by IBD frequently experience periods of acute illness, sometimes requiring hospitalization, which has been associated with a significant deterioration to health-related quality of life (HRQoL) (Chang, 2020; Knowles et al., 2018a,b; Mikocka-Walus et al., 2020). The disease is estimated to affect 6.8 million individuals globally, with around 653 per 100,000 Australians estimated to be living with IBD (GBD 2017 Inflammatory Bowel Disease Collaborators, 2020; Busingye et al., 2021). Prevalence rates of IBD in western regions are predicted to reach 1.0% by 2030, representing an almost doubling in prevalence over 20 years (Kaplan & Windsor, 2021).

While managing the physical symptoms is often the focus of treatment, research findings highlight that individuals with IBD are also at increased risk of experiencing poor psychological wellbeing (Gao et al., 2021; Mikocka-Walus et al., 2016). Emerging evidence supports the existence of a gut–brain link and suggests that this bi-directional link plays a role in the pathophysiology of IBD (Gracie et al., 2018). In a systematic review and meta-analysis conducted by Barberio et al. (2021) (*n* = 30,118), pooled prevalence of anxiety and depressive symptoms was found to be 32.1% and 25.2%, respectively, for individuals with IBD. Further, a large retrospective cohort study conducted in the United States by Tarar et al. (2022) (*n* = 963,619) found the prevalence of anxiety and depression to be significantly higher in individuals with IBD when compared to those without the condition (20.9% vs. 15.0% and 16.9% vs. 12.3%, respectively). The functional impairment associated with the unpredictability of symptom fluctuations, financial pressures, and the absence of a cure is thought to adversely affect the psychological wellbeing of individuals living with IBD (Gao et al., 2021; Knowles et al., 2018a,b). Individuals with IBD describe their experience as a “vicious cycle,” with IBD affecting their social, psychological, and physical functioning (Mikocka-Walus et al., 2021). Themes of disease-specific distress reported by those living with IBD include “living with uncertainty” and “not being believed” (Woodward et al., 2016).

Individuals with IBD who experience comorbid psychological distress may be less likely to adhere to treatment regimens or proactively seek necessary healthcare, worsening the course of the disease (Lewis et al., 2019). Previous qualitative research highlights some individuals perceived their IBD experience to be “hopeless,” especially when experiencing depressive symptoms, and were therefore more likely to disengage from health services (Mikocka-Walus et al., 2021). A 22-month longitudinal study by Kochar et al. (2018) (*n* = 4314) found that patients with IBD were at higher risk of relapse and aggressive disease if they also experienced higher levels of baseline depression.

Evidence for a brain–gut link in IBD has sparked advocacy for the inclusion of psychological support into treatment plans to address the disease through a biopsychosocial lens (Kiebles et al., 2010). Despite this, access to psychological support remains sparse or poorly directed for those with IBD, with the focus of treatment remaining predominantly on biomedical intervention (Kiebles et al., 2010). A cross-sectional survey conducted by Crohn’s & Colitis Australia (2018) (*n* = 731) found that while most individuals with IBD reported having access to gastroenterologists, only 12% of participants had access to psychologists, with requests for access to the wider multi-disciplinary team a common theme among participants. Current evidence around the efficacy of different types of psychotherapy is mixed and there remain questions around what types of support might be most effective in improving patient outcomes for those with IBD (Ballou & Keefer, 2017; Gracie et al., 2017; Knowles et al., 2013). Individuals with comorbid psychological distress have been largely overlooked in previous studies investigating psychotherapy interventions for those with IBD, highlighting a gap in the literature.

Cognitive-behavioral therapy (CBT), a psychotherapy which aims to identify, challenge, and restructure problematic automatic thoughts to reduce symptoms of psychological distress, has been a major focus of psychological interventions for IBD (Bennebroek Evertsz et al., 2017; Gregory, 2019). CBT is demonstrated to be somewhat effective in improving HRQoL and depressive symptoms for those with IBD; however, there remain mixed reviews on its long-term efficacy and its effectiveness, if any, in treating anxiety symptoms (Ballou & Keefer, 2017; Bennebroek Evertsz et al., 2017; Gracie et al., 2017; Hanlon et al., 2018; Mikocka-Walus et al., 2017; Stapersma et al., 2020). Qualitative research has identified that strategies targeting behavioral responses to stress and emotion may be helpful in reducing physical symptoms for those with IBD (Fawson et al., 2022; Mikocka-Walus et al., 2021). Despite this, there are limited and inconclusive research findings regarding the perspectives of those living with IBD, including what types of psychotherapy they feel might be most useful for managing the physical and psychological consequences of IBD and why.

Acceptance and commitment therapy (ACT), a derivative of CBT, is thought to be particularly helpful in increasing functionality for those living with chronic illness, as it promotes mindful acceptance of unwanted internal experiences, while emphasizing value-directed goal setting and decision-making (Hayes et al., 2012). ACT is demonstrated to reduce psychological distress in a range of chronic health conditions such as chronic pain and diabetes, possibly due to shared characteristics of unpredictability, lack of cure, and related negative internal experiences (Brassington et al., 2016). Preliminary findings from a recent randomized controlled trial (RCT) show ACT may be effective in reducing levels of depression and improving HRQoL ratings in individuals with IBD when compared to treatment as usual (Wynne et al., 2019). In particular, previous qualitative research demonstrates individuals living with IBD find the acceptance aspect of ACT helpful in reducing their mental load, allowing them to get the most out of their life despite the chronic nature of the condition (Mikocka-Walus et al., 2021).

Research exploring the most effective *modes* of psychological treatment delivery for those living with IBD remains a priority, particularly given the potential barrier physical symptoms may pose to accessing in-person support and the increased delivery of psychotherapy via telehealth since the COVID-19 pandemic. Online versus in-person delivery of psychotherapy have produced inconclusive results (Lavelle et al., 2022; Mikocka-Walus et al., 2017), whereas the combination of self- and therapist-led online psychotherapy programs appears to be most beneficial to this population (Dober et al., 2021; Mikocka-Walus et al., 2017). Despite this, exploration of perspectives around hybrid therapist- and self-directed online delivery, including what individuals with IBD regard as the most helpful aspects, is missing from the current literature.

## Objective

The current qualitative study was conducted alongside a quantitative feasibility RCT (Romano et al., 2023) and aims to understand the experiences of people with IBD and comorbid anxiety/depression undertaking a novel hybrid ACT intervention (therapist sessions and self-directed therapy), compared to those undertaking IBD psychoeducation. We were specifically interested in understanding (1) what participants thought was meaningful and useful to their lives from the hybrid ACT intervention; and (2) how they experienced recorded versus telehealth materials, compared to the active control.

## Method

### Ethics Approval

Ethics approval was obtained from the Deakin University Research Ethics Committee in September 2021 (Ref. 2021-263) and the New Zealand Central Health and Disability Ethics Committee in December 2021 (Ref. 2021 EXP 11384). The registration number associated with the trial is ACTRN12621001316897.

### Design

This qualitative study was conducted parallel to a randomized controlled feasibility trial, comparing ACTforIBD (*n* = 31) to active control (*n* = 31). Quantitative outcomes assessed in the trial included recruitment and retention rates, a satisfaction scale, and changes on distress and disease related outcomes. The present study focuses on the lived experience of participants, using data collected during in-depth semi-structured interviews conducted at the conclusion of the 8-week intervention, and 3 months following the intervention. Study design adhered to the COREQ checklist outlined by Tong et al. (2007) and further information can be found in the published protocol for quantitative feasibility study (Evans et al., 2022). Participants in the experimental group took part in the ACTforIBD program, while the ActiveControl group completed a psychoeducation-focused program adapted to IBD.

Qualitative methodology was guided by a phenomenological, or lived experience, framework. Phenomenology is helpful in exploring whether an intervention is acceptable to participants, as well as how or why an intervention might be effective (Smith, 2008). The existence of an unobservable “true” reality in the data was assumed using a critical realist approach and it is therefore acknowledged that data analysis cannot be independent of the author’s perspective (Braun & Clarke, 2013).

### Participants

The sample consisted of ten participants each from the experimental and ActiveControl groups (*n* = 20). Participants were the first ten participants from the feasibility trial from each group to agree to take part in the interviews. Recruitment took place via study advertisements posted to social media which directed participants to express their interest in participating in the study either via an online form or by contacting the research team by e-mail. A plain language statement was supplied to participants and written consent was obtained prior to commencing the study. Participants completed the Kessler Psychological Distress Scale (K10) (Kessler et al., 2002) and were asked to provide evidence of IBD diagnosis for trial managers to assess eligibility. Inclusion criteria included the following: (1) IBD diagnosis, (2) mild to moderate psychological distress, as indicated by a score of between 16 and 29 on the K10, (3) age of 18 years or above, (4) residing in Australia or New Zealand, (5) ability to write and read in English, and (6) Internet access. Exclusion criteria included severe levels of psychological distress (scores of 30–50 on K10) or no psychological distress (scores of less than 16 on the K10), consistent with guidelines on K10 cut-scores (Andrews & Slade, 2001). Baseline sociodemographic data were collected prior to randomization (stratified based on IBD activity) via Qualtrics into one of the two study groups, with ten participants in each group (Table 1).

**Table 1:** Demographic data of individuals with IBD, *n* = 20

Mean (*SD*); number (%)	ACTforIBD (*n* = 10)	ActiveControl (*n* = 10)
Age	33.7 (*10.9*)	32.5 (*7.6*)
Gender (female)	9 (90.0%)	9 (90.0%)
Language spoken at home		
English	9 (90.0%)	10 (100.0%)
Other	1 (10.0%)	–
Diagnosis	UC (30%)	UC (70%)
	Crohn’s (70%)	Crohn’s (30%)
IBD activity (Manitoba Index)		
High	10 (100.0%)	8 (80.0%)
Low	–	2 (20.0%)
Psychological distress (K10)		
Mild	6 (60.0%)	7 (70.0%)
Moderate	4 (40.0%)	3 (30.0%)
Highest level of education obtained		
Year 12 or equivalent	3 (30.0%)	–
TAFE	–	1 (10.0%)
University degree (Bachelor/Master)	7 (70.0%)	7 (70.0%)
Other	–	2 (20.0%)
Marital status		
Single	6 (60.0%)	3 (30.0%)
Married/de facto	4 (40.0%)	7 (70.0%)
Paid employment status		
Employed full time	7 (70.0%)	6 (60.0%)
Employed part time	1 (10.0%)	4 (40.0%)
Student	1 (10.0%)	–
Retired	1 (10.0%)	–

### Intervention and ActiveControl Condition

Topics covered in both the intervention and control programs are shown in Table 2. Both conditions involved 8 weeks of individual treatment, conducted online.

**Table 2:** The ACTforIBD and active control modules

ACTforIBD modules	Active control modules
Module 1: Commitment to therapy and assessment	Module 1: Stress management
Module 2: Creative hopelessness	Module 2: Progressive muscle relaxation
Module 3: Personal values	Module 3: Problem solving
Module 4: Mindfulness	Module 4: Coping strategies
Module 5: Fusion and defusion	Module 5: How to think more assertively
Module 6: Acceptance	Module 6: How to behave more assertively
Module 7: Values and goals	Module 7: Sleeping for better wellbeing
Module 8: Commitment and overcoming barriers moving forward	Module 8: Towards a healthy self-esteem

The ***ACTforIBD program*** was based on principles of ACT such as mindfulness, value-based decision-making, and defusion and was co-designed with consumers (Dober et al., 2021; Keeton et al., 2015). It included eight fully scripted modules designed to occur weekly as 1-h sessions. The program was a mix of therapist- and self-directed sessions, with the first three sessions and the last session supported by a provisionally registered psychologist via telehealth (individual zoom sessions), under the supervision of a fully registered clinical psychologist. The sessions in between were self-directed and included the use of video content plus self-assessment activities.

The ***ActiveControl group*** took part in a psychoeducation-focused program and mirrored the duration and demands of the experimental group. The program ran for 8 weeks, with 1 h of involvement with the website by participants recommended per week of the program. Therapist-led sessions occurred via telehealth during the first 3 weeks of the program and the last week (individual zoom sessions), in line with the experimental group; however, therapists focused on psychoeducational content rather than therapeutic engagement.

### Measures

Sociodemographic data collected included the following: age, gender, education level, employment and marital status, language spoken at home, and postcode. Principles from the most significant change (MSC) technique (Davies, 2005) were used to develop the interview questions. Participants were asked about their most significant changes, why these changes were significant and what meaning participants derived from these changes. Interview questions also covered perceived barriers and enablers to undertaking the program (i.e., What kept you motivated, or supported you, to continue taking part in the program?) and which elements of the program participants continued to use (i.e., What parts of the ACT intervention have you continued to use in the last 3 months?) (see Appendix). Interviews were conducted immediately post-intervention, and again at 3 months post-intervention. Forty-five minutes was allocated for interviews.

### Data Analysis

Data familiarity was achieved following several read-throughs of transcribed interviews. The interview transcripts were analyzed using reflexive thematic analysis following the six-step process outlined by Braun and Clarke (2020), namely: (1) familiarization with the data, (2) initial coding of the data, (3) initial interpretation of themes, (4) revision of themes, (5) further refining and naming of themes, and (6) reporting on produced themes. The first author (a registered nurse and clinical psychology student) completed all steps, including coding all interview transcripts. Coding was discussed with the qualitative team (KW, SE, AMW), and themes were revised and agreed upon by the team. NVIVO software (released in March 2020) was used during analysis. Researchers used deductive and inductive thematic analysis to allow themes to represent participants’ reflections on the use of ACT (Braun & Clarke, 2020). Transcribed interview data were interpreted separately for each group, given the aim of the study was to explore the perspectives of an ACT intervention in comparison to an ActiveControl group.

In terms of reflexivity, the qualitative analysis team comprised people with both insider and outsider perspectives, including those with lived IBD experience, nursing, clinical psychology, and health psychology research experience. Bracketing was used during data collection and interpretation to set aside assumptions, vested interests and personal beliefs stemming from prior experience and knowledge of authors who work with IBD populations; both in research and tertiary healthcare systems (Fischer, 2009). While the design of the experimental intervention utilized in this feasibility study was designed in tandem with authors on this paper, IBD research was novel to both the interviewer and first author, attenuating influence of prior investments on results.

## Results

Participant demographics for both groups are contrasted in Table 1. Participants were aged between 21 and 58 years (*M* = 33.1, SD = 9.2), with majority of participants identifying as female (90.0%). In the ACTforIBD group, the majority of participants were diagnosed with Crohn’s, while the majority of the control group were diagnosed with Ulcerative Colitis. Predominantly, English was the language spoken at home by participants (95.0%). Over half of participants were university educated (70.0%) and employed full time (65.0%), 25.0% were employed part-time and 10.0% of participants were either studying or retired. Over half of participants reported mild psychological distress (65.0%) as opposed to moderate psychological distress (35.0%), and 90.0% reported high IBD activity. Demographic spread between both groups was comparable. All participants in the control group completed 100% of sessions (therapist-led and self-directed). Seven of the ten participants in ACTforIBD completed 100% of sessions (two participants completed 7/8 sessions, and one completed 5/8 sessions).

Three main themes were identified through reflexive thematic analysis which were shared by both the ACT and ActiveControl groups: *I Am Worth Advocating For*, *Present Moment is My Biggest Ally*, and *Ambivalence About Self-Directed Modules*. Two main themes interpreted from the ACTforIBD group included the following: *Moving Toward Values* and *Symptoms Are Going to Happen* while two main themes interpreted from the ActiveControl group included the following: *It’s Ok to Say No* and *Reset and Refresh*. All themes and subthemes are displayed in Table 3.

**Table 3:** Themes and subthemes interpreted through reflexive thematic analysis

	Themes	Sub-themes
Shared	I Am Worth Advocating For	Visibility for IBD Self-Compassion
	Present Moment Is My Biggest Ally	Thoughts Are Not Reality Mind–Body Connection
	Ambivalence About Self-Directed Modules	
ACTforIBD	Moving Toward Values	Adjusting Focus to What’s Important
	Symptoms Are Going to Happen	Acceptance of Negative Thoughts and Emotions
ActiveControl	It’s Ok to Say No	Assertiveness and Self-Esteem
	Reset and Refresh	Affirming Useful Coping Strategies

### Shared Themes

#### Theme One: I Am Worth Advocating For

##### Visibility for IBD

Participants felt the very fact that research was being conducted into psychological wellbeing in IBD meant there were people in similar situations and that their experience mattered. For many, this was the first time they had been exposed to individualized psychological support which specifically targeted IBD and this was hugely validating, especially for some participants whose experience of psychological support prior was perceived as suboptimal.

“…actually all the feelings and emotions and challenges that I’m experiencing as a result of this disease are really valid and really real.” (P6, ActiveControl, 3-month follow-up, 27-year-old woman)

Participants considered the provisionally registered psychologists personable, knowledgeable in IBD and felt the intervention was provided in a person-centered manner, improving both their understanding of content, and increasing motivation to continue using strategies beyond the 8-week program. There was a perception that the addition of connections with peer support groups would help with program engagement by growing a long-term support network of people with an understanding of their situation:

“…if there was an opportunity to get together with people and do it like on a one day with other people in the room, so you’re able to share experiences and discuss things a little bit more.” (P9, ACTforIBD, 3-month follow-up, 41-year-old woman)

##### Self-Compassion

At the 3-month mark, participants reported an increased focus on self-compassion in their day-to-day life, regardless of whether they completed the ACTforIBD or ActiveControl program. Commencing the program, participants reported feeling internalized shame resulting from societal stigma around IBD, and therefore found the IBD-specific content validating and helpful in attenuating this shame:

“It’s made me think about the diagnosis a lot differently and be like, Well, actually, it’s not your fault.” (P9, ActiveControl, post-intervention, 39-year-old woman)

Identifying this shame and being provided with acknowledgment of their challenges with IBD allowed participants to turn inwards and focus on their own wellbeing, which resulted in reduced levels of perceived stress:

“…definitely one of the big parts for me was recognising the need for self-compassion and that it hasn’t previously been there” (P5, ACTforIBD, 3-month follow-up, 25-year-old woman)

#### Theme Two: Present Moment Is My Biggest Ally

##### Thoughts Are Not Reality

All participants reported an increased awareness of negative thought patterns 3-months post program completion, and that this awareness was enough to reduce their impact on perceived stress levels. Many participants felt this enabled them to focus on the present moment, rather than perpetuate a cycle of negative thoughts which would normally lead to increased stress levels for them. One participant reported they were now able to label their worry as either “good worry” or “dirty worry,” which reduced the power unhelpful thoughts had over their stress:

“I just like, hold on. This is a thought….and recognising it’s that rather than taking it as a fact.” (P6, ACTforIBD, post-intervention, 41-year-old woman)

##### Mind–Body Connection

Participants from both groups indicated an important takeaway was increased understanding of the interplay between stress levels and physical IBD symptoms following the program:

“I think overall acknowledging that, like making that link between my flare up being because I was stressed.” (P7, ActiveControl, 3-month follow-up, 28-year-old woman)

Participants from both groups reported that being more present was effective in reducing stress at the 3-month mark. The ACTforIBD group described the present moment as making the most of what they had, with one participant reflecting during their 3-month follow-up interview that they had previously made a “cognitive choice” to tune out of body sensations because they were “too overwhelming.” However, following the ACTforIBD intervention, they were able to recognize that this disassociation was negatively affecting all parts of their life. They described the process of having to “relearn” connections between physical and mental experiences a significant takeaway from the intervention (P5, ACTforIBD, 3-month follow-up, 25-year-old woman). Participants from the ActiveControl group found mindfulness techniques provided practical coping strategies in moments of stress. One participant mentioned using breathing techniques during the drive home from work or before medical appointments to reduce stress while another reported finding progressive muscle relaxation techniques useful:

“Particularly if I’m doing computer work… And I’m feeling tense in my muscles then I’ll do that progressive muscle. Because I really like that, it was a really positive way of dealing with that tension.” (P8, ActiveControl, 3-month follow-up, 45-year-old woman)

#### Theme Three: Ambivalence About Self-Directed Modules

Participants from both groups reported neglecting self-directed modules at times due to lack of accountability, unlike the face-to-face modules where they felt accountable to the provisionally registered psychologist.

“…sometimes the online ones I’d find harder to get through, I guess just because, I think you focus more when it’s a real person, I suppose, just naturally.” (P7, ACTforIBD, 3-month follow-up, 27-year-old woman)

Many participants reported usability and technical issues such as the inability to save progress or reflect on answers provided within modules at a later timepoint, making it difficult for them to gauge their progress when working alone. Participants currently experiencing flare ups reported this as a barrier to completing self-directed modules and continuing to use strategies generally. The ACTforIBD group was particularly challenged by the self-directed content as it was new to them and reported wanting to ask questions of clinicians to clarify understanding.

Conversely, some participants felt the self-directed delivery was particularly useful and appreciated the flexibility it afforded. Although findings leaned toward usability challenges with the self-directed modules, the experiences of those who enjoyed these modules are also noteworthy:

“…the self-directed learning was wonderful, cause that’s something. Yeah, you can prioritize your time your own way.” (P1, ActiveControl, 3-month follow-up, 24-year-old woman).

### ACTforIBD Themes

#### Theme One: Moving Towards Values

##### Adjusting Focus to What’s Important

This group found decision-making according to their values a takeaway from the ACT intervention at the 3-month mark:

“I can live, like a life like that moves towards my values…despite having Crohn’s” (P3, ACTforIBD, 3-month follow-up, 22-year-old woman)

For many, this was their first exposure to values guided decision-making, and they found this change to their decision-making ability helpful in countering stress:

“I just think that, yeah, they’re (values) really important to actually change behaviour and motivation” (P2, ACTforIBD, post-intervention, 34-year-old woman)

One participant (P9, ACTforIBD, 3-month follow-up, 41-year-old woman) identified through the ACTforIBD intervention that a personal value of theirs was “helping others” and reported during the 3-month interviews that they had cut back work in order to volunteer, leading to increased feelings of wellbeing.

#### Theme Two: Symptoms Are Going to Happen

##### Acceptance of Negative Thoughts and Emotions

Participants perceived the notion of accepting all thoughts and emotions relating to their IBD particularly helpful in reducing their mental distress. They described acceptance as more of an abstract concept they were constantly working on, which was foundational to their everyday outlook, rather than a specific strategy to be drawn on in moments of acute stress. For this reason, many reflected that ACT was a worthwhile modality for use in IBD given the often uncomfortable and inescapable reality of living with the condition. Acceptance of negative thoughts and emotions enabled participants to focus on what was important to them, rather than dwell on the negative aspects of their condition, which may have been their usual coping mechanism:

“These symptoms are going to happen, it doesn’t necessarily mean that you’re going to end up, you know, really sick or anything.” (P4, ACTforIBD, 3-month follow-up, 30-year-old woman)

One participant (P5, ACTforIBD, 3-month follow-up, 25-year-old woman) described previously feeling frustrated at the cost of her IBD medication, which often led to non-compliance and worsening of symptoms. Three months following the ACT intervention, she reported coming to terms with the fact that it was ok to “be disappointed,” to “grieve” parts of her life, and accept that she needed the medication, despite the cost.

Participants reflected on the way learning ACTforIBD skills would have been helpful early in their IBD journey, with one participant noting that it would be a valuable program for children and adolescents:

“I think for teenagers or children, they get diagnosed, I reckon that would be a great target for people.” (P9, ACTforIBD, post-intervention, 41-year old woman)

### ActiveControl Themes

#### Theme One: It’s Ok to Say No

Modules from the ActiveControl included **Assertiveness and Self-Esteem** which participants believed enabled them to advocate for themselves and gain a more balanced perspective around their IBD at the 3-month mark. Some participants described making changes in their workplace to reduce stress following these modules, such as flagging their ideas to managers or setting boundaries around work hours. One participant, when asked what their biggest takeaway from the self-esteem content was, replied,

“It’s changed my perception of myself, which makes it easier for me to cope” (P9, ActiveControl, post-intervention, 39-year-old woman)

#### Theme Two: Reset and Refresh

##### Affirming Useful Coping Strategies

Modules around sleep, stress management, and muscle relaxation included in the ActiveControl were commonly reported as content participants had seen before. Many participants felt this was a good refresher and were able to acknowledge they had developed healthy coping mechanisms, some without realizing it, resulting in a sense of pride. One participant reported they gave themselves a “pat on the back” for having previously implemented strategies presented in modules to improve sleep (P1, ActiveControl, post-intervention, 24-year-old woman). Some participants in the ActiveControl group found nothing new of interest in the intervention, having been exposed to similar material in the past and this was a barrier to them perceiving any benefits.

## Discussion

This study explored the experience of a hybrid ACT intervention for increasing psychological wellbeing from the perspectives of individuals living with IBD with comorbid symptoms of anxiety and/or depression, in comparison with an ActiveControl group.

Reflexive thematic analysis revealed themes shared by participants from both groups: *I Am Worth Advocating For*, *Present Moment Is My Biggest Ally*, and *Ambivalence About Self-Directed Modules*. Participants in the ACTforIBD intervention felt concepts learnt in the program including *Moving Toward Values* and acceptance that *Symptoms Are Going to Happen* were effective in reducing perceived levels of psychological distress. Thus, participants in ACTforIBD found the psychological processes of self-compassion, mindfulness, values, and acceptance to be the most helpful aspects of ACT. Participants in the ActiveControl group felt the psychoeducation-focused content provided an opportunity to *Reset and Refresh* and empowered them to reduce their stress with the perceived notion that *It’s Ok to Say No*. Captured by the data were subtle behavior changes as perceived by participants including: increased use of mindfulness techniques, improved decision-making ability, feeling more comfortable in the present moment, and responding with self-compassion.

Feelings of shame reported by participants around their IBD are consistent with previous qualitative research which demonstrates that individuals with IBD often experience a “feeling of otherness,” resulting in them feeling they must suffer in silence (Mikocka-Walus et al., 2021; Muse et al., 2021). Given participants in this study were living with comorbid symptoms of anxiety and/or depression, mental health stigma may have compounded their feelings of isolation before engaging in the current study and may explain why self-compassion and visibility for IBD were such salient themes among participants’ perspectives. Ongoing advocacy for psychological treatment in IBD, oftentimes overlooked in favor of a biomedical approach, is further supported by the perspectives of individuals in this study, who felt simply having their experience validated by the provisionally registered psychologist was enough to positively impact their psychological distress (Davis et al., 2020; Mikocka-Walus et al., 2020; Tarar et al., 2022). Perspectives shared by participants in the current study that an IBD-specific psychological intervention would be particularly valuable close to time of diagnosis was congruent with past research and may be useful in informing best practice models of care (Dober et al., 2021; Fawson et al., 2022; Feeney et al., 2022).

The shared view by participants from both groups that being present was helpful in coping with stress was particularly interesting, given mindfulness in ACTforIBD focuses on *acceptance* of negative experience as a gateway to greater HRQoL, whereas the ActiveControl program offered mindfulness as an *appraisal of* and distraction from negative experience. Both groups shared an emphasis on understanding the bi-directional brain–gut link in IBD, which may explain why mindfulness was so well received across both groups, even if the specific technique of engaging in mindfulness differed. Mindfulness-based interventions for IBD have been well received by participants in past research as effective “tools” to draw on in stressful situations (Cebolla et al., 2021) and this sentiment was shared by participants in the ActiveControl group. In contrast, participants in the ACTforIBD intervention felt mindfulness, in the form of accepting negative experiences in the present moment, resulted in an enduring and positive change to their everyday outlook. Previous qualitative research looking at ACT interventions for IBD proposes that *person-centered* delivery of mindfulness techniques ensures optimal engagement from individuals from a wide demographic background (Dober et al., 2021). In line with this, the positive uptake of mindfulness in the current study may be related to the reflection by participants that interventions overall were person-centered.

Many in the ACTforIBD intervention found acceptance of negative thoughts and emotions associated with IBD an effective strategy in attenuating distress and this was congruent with findings from a small cross-sectional study by Kiebles et al. (2010) (*n* = 38), which demonstrated a positive correlation between symptom tolerance and less perceived stress and greater socioemotional functionality. Previous qualitative research exploring perspectives of those living with IBD has identified that aspects of the condition that are outside of an individual’s control are considered anxiety-inducing, and this may indicate why acceptance modules were so well received in the current study (Karadag et al., 2022). Consistent with the qualitative exploration of ACT as an intervention for IBD conducted by Dober et al. (2021), participants in the ACTforIBD intervention found the experience of connecting to their physical sensations, thoughts, and emotions in the present moment a confronting, yet worthwhile process in reducing psychological distress. The same was demonstrated in an RCT by Wynne et al. (2019) where stress and depressive symptoms decreased significantly following an 8-week ACT intervention which targeted stress, despite external stressors remaining the same for participants. Findings from the current study around the perceived effectiveness of acceptance of negative experience further supports the use of ACT in IBD populations, given this is a core principle of the modality (Hayes et al., 2012).

Committed action which aligns with personal values is another core feature of ACT, and this was reflected in the perspectives of participants in this group as a practical takeaway to reduce distress. The use of personal values as a navigational tool aims to empower individuals to confront avoidant behavior, a common coping strategy in IBD given the nature of symptoms, which often leads to worsened outcomes in all aspects of an individual’s everyday life (Volpato et al., 2021). A systematic review by Hayes et al. (2020) (*n* = 918), found that behavioral disengagement and decreased activity, as a coping mechanism in IBD, negatively influenced psychological distress. Similarly, a cross-sectional study by Kantidakis et al. (2021) (*n* = 261) found that maladaptive coping mechanisms partially mediated the relationship between illness perception and psychological distress in individuals with IBD, highlighting the importance of empowering individuals to engage in effective coping strategies and reduce their risk of psychological distress.

The rationale for online blended delivery of psychological interventions, such as those in the current study, is to bridge barriers to accessing psychological support such as unpredictable symptoms and lack of resources including appropriately trained staff (Dober et al., 2021; Forbes & Johnson, 2021; Mikocka-Walus et al., 2020). Previous qualitative research which explored perspectives of both consumers and health professionals found the inclusion of both therapist-led and self-directed modules maximizes exposure to subject matter experts while circumnavigating possible non-adherence with fully self-directed programs (Dober et al., 2021; Hanlon et al., 2022, Mikocka-Walus et al., 2017). While many participants in the current study reported feeling a lack of motivation to complete self-directed modules, others felt self-directed modules gave them the flexibility to complete modules around their schedules. Providing additional external links within intervention material to empower individuals to source further information independently may address challenges experienced by some participants in the ACTforIBD group, who felt a need to clarify understanding with their psychologists during self-directed modules (Fawson et al., 2022).

Perspectives of participants in the current study around the need for social support as part of psychotherapy may be important in informing future interventions; however, past literature shows individuals with IBD have mixed views on peer group engagement. Some research suggests social networking may be useful in reducing feelings of social isolation, while some research suggests that individuals with IBD feel exposing themselves to others’ experiences of IBD, especially if perceived as worse than their own, may induce anxiety (Dober et al., 2021; Ewais et al., 2020; Karadag et al., 2022). A qualitative study by Karadag et al. (2022) found IBD support groups on social media increased participants’ access to relevant information around IBD and, alongside psychotherapy, this type of support may be important for a population that often feels isolated. Further qualitative exploration of social connection in tandem with psychological intervention may therefore be required to inform future practice.

Similar information to that provided in the psychoeducation intervention is available on websites such as Crohn’s & Colitis (Australia and New Zealand) and may explain why some participants in the ActiveControl group felt the program was a good refresher. The reflection that coping strategies learnt in the ActiveControl program were easily actionable aligns with the philosophy of CBT, which aims to empower individuals to change maladaptive behaviors or thoughts in the here and now, in a structured, time-limited manner (Fenn & Byrne, 2013). Feelings of powerlessness or shame are commonly associated with a diagnosis of IBD, resulting from unpredictability of symptoms and societal stigma, and this may be why modules on assertiveness and self-esteem were so well received by this group (Volpato et al., 2021). A cross-sectional study by Opheim et al. (2020) (*n* = 411) demonstrated a significant association between low self-esteem and psychological distress in individuals with IBD, supporting perspectives of those in the current study that interventions targeting self-esteem might be useful in optimizing IBD management.

## Strengths and Limitations

This study shed light on the experiences of those with IBD and symptoms of comorbid anxiety and/or depression, following the delivery of an ACT intervention, which has not been addressed previously. The experience of blended online delivery of an ACT intervention, using both therapist- and self-directed modules, was assessed in the current study, providing valuable insight into barriers and enablers to accessing psychological support for those with IBD and symptoms of psychological distress. The use of an active control group to comparatively explore individual perspectives on an ACT intervention for IBD is novel and a strength of the study.

This study captured the voices of highly educated individuals, who were fully employed, predominantly spoke English at home and were likely highly tech savvy. Thus, perspectives explored may not be fully representative of the diversity within the IBD population. Additionally, there were relatively few individuals who identified as male or non-binary in this sample, which was a limitation of this study. Individuals who identify as male have expressed hesitation around psychotherapy and help-seeking for psychological distress in past literature, including those with IBD (Dober et al., 2021; Seidler et al., 2018). Future research should therefore aim to explore perspectives of individuals from outside of this group, representing diverse gender and cultural identities, geographical locations, and educational levels.

## Conclusion

The effective delivery of psychotherapy, informed by the voices of those with the condition, is imperative for individuals with IBD given their increased risk of experiencing comorbid psychological distress. Overall, a focus on validation of experience, self-compassion, and the mind–body connection may be particularly useful when delivering psychotherapy to these individuals. Acceptance of negative experiences and commitment to valued action as part of the ACTforIBD intervention may be particularly useful in this population for improving psychological wellbeing, given the inescapable nature of the condition. Psychoeducation-based programs may provide those with IBD with a good refresher of effective coping strategies while certain aspects, especially those relating to self-esteem and assertiveness, may be useful to enhance future ACT interventions. Blended therapist-led and self-directed program delivery bridges gaps for a population that faces unique barriers to accessing mental health care and was mostly well received in the current study, however, may not be a good fit for all. Links to external resources to support users through self-led modules and peer networking opportunities may increase engagement with content long term.

## Data Availability

The data that support the findings of this study may be available on request from the corresponding author. The data are not publicly available due to privacy or ethical restrictions.

## Appendix

### Semi-structured Interview Questions

1. What brought you to take part in the ACT intervention?
2. What kept you motivated, or supported you, to continue taking part in the program?
3. What did you find were the most challenging aspects of taking part in the program?
4. What did you like most (find most helpful) about the program?
5. What did you like least (find least helpful) about the program?
6. How could we improve the content?
7. How could we improve the way it is delivered?

### Additional Questions Asked at 3-Month Follow-Up

8. What have been the most significant changes experienced over time regarding mental health that you would attribute to ACT?
9. What have been the most significant changes experienced over time regarding physical health that you would attribute to ACT?
10. What makes these changes significant?
11. What would you say to a friend interested in taking part?
12. Anything else you would like to share about your experiences?
13. What parts of the ACT intervention have you continued to use in the last 3 months?
14. How often do you think you would use strategies learned from ACT? Can you give me an example of where you might implement these strategies?

## Code Availability

N/A.
